# Divide and conquer: Multicolonial structure, nestmate recognition, and antagonistic behaviors in dense populations of the invasive ant *Brachymyrmex patagonicus*

**DOI:** 10.1002/ece3.7396

**Published:** 2021-03-18

**Authors:** Pierre‐André Eyer, Phillip T. Shults, Madeleine R. Chura, Megan N. Moran, Morgan N. Thompson, Anjel M. Helms, Raj K. Saran, Edward L. Vargo

**Affiliations:** ^1^ Department of Entomology Texas A&M University College Station TX USA; ^2^ Bayer Environmental Sciences College Station TX USA

**Keywords:** Ants, Colony breeding system, Invasive species, Monogyne, Cuticular hydrocarbons, Aggression

## Abstract

The ecological success of ants has made them abundant in most environments, yet inter‐ and intraspecific competition usually limit nest density for a given population. Most invasive ant populations circumvent this limitation through a supercolonial structure, eliminating intraspecific competition through a loss of nestmate recognition and lack of aggression toward non‐nestmates. Native to South America, *Brachymyrmex patagonicus* has recently invaded many locations worldwide, with invasive populations described as extremely large and dense. Yet, in contrast with most invasive ants, this species exhibits a multicolonial structure, whereby each colony occupies a single nest. Here, we investigated the interplay between genetic diversity, chemical recognition, and aggressive behaviors in an invasive population of *B. patagonicus*. We found that, in its invasive range, this species reaches a high nest density with individual colonies located every 2.5 m and that colony boundaries are maintained through aggression toward non‐nestmates. This recognition and antagonism toward non‐nestmates is mediated by chemical differentiation between colonies, as different colonies exhibit distinct chemical profiles. We highlighted that the level of aggression between colonies is correlated with their degree of genetic difference, but not their overall chemical differentiation. This may suggest that only a few chemical compounds influence nestmate recognition in this species or that weak chemical differences are sufficient to elicit aggression. Overall, this study demonstrates that invasive ant populations can reach high densities despite a multicolonial structure with strong aggression between colonies, raising questions about the factors underlying their ecological success and mitigating negative consequences of competitive interactions.

## Graphical abstract

Invasive ants usually form large supercolonies, in which aggression between non‐nestmates is therefore absent due to a loss of a functional chemical recognition system. Here, we investigated the interplay between genetic diversity, chemical recognition, and aggressive behaviors in an invasive population of *Brachymyrmex patagonicus*, an invasive ant species that surprisingly does not form supercolony. We found a high nest density despite the maintenance of colony boundaries through aggression toward non‐nestmates. This recognition and antagonism toward non‐nestmates are mediated the upkeep of chemical differentiation between colonies, with the level of aggression between colonies is correlated with their degree of genetic difference.

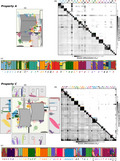

## 1 INTRODUCTION

Reproductive division of labor is the hallmark of social insects, whereby nonreproductive workers forego their own reproduction to perform diverse colony tasks, while reproduction is carried out by a single, or few individuals within colonies (Hamilton, 1964). The evolutionary stability of this system requires high relatedness within colonies, enabling nonreproducing workers to achieve indirect fitness by enhancing the reproduction of kin through altruistic behaviors. Therefore, it typically demands strict colony boundaries to preserve significant relatedness within colonies, assuring altruistic behaviors performed by workers benefit related individuals.

To maintain distinct colonies, insect societies rely on well‐developed nestmate recognition systems, which prevent or reduce intraspecific and interspecific invaders from entering the colony (i.e., competitors, enemies, or parasites) (Hölldobler & Wilson, 1990). Outside of the physical boundaries of the nest, nestmate recognition also allows for discrimination of nestmates and non‐nestmates to prevent antagonism between nestmate foragers or allow a colony to monopolize resources by ousting non‐nestmates. Social insects primarily use chemical cues to discriminate between nestmates and alien conspecifics. These are mainly blends of long‐chain hydrocarbons found on the insect cuticle (Howard & Blomquist, 2005; Lahav et al., 1999; Wagner et al., 2000). The relative proportion of each compound within the blend generally differs between colonies within species, whereas distinct species carry different compounds (Bos & d'Ettorre, 2012; Sturgis & Gordon, 2012; Van Zweden et al., 2010). Cuticular hydrocarbon (CHC) blends are influenced by both genetic and environmental factors, such as nest material or diet (Lenoir et al., 2001; Liang & Silverman, 2000; Walsh et al., 2020). Consequently, the similarity among nestmates is first established by their shared genetic background. It is later reinforced by living in a common nest, frequent contacts between nestmates through allogrooming, and their recurrent exchanges through trophallaxis (Dahbi et al., 1999; Hölldobler & Wilson, 1990). Generally, differences in CHC blends between colonies determine the level of aggression displayed in intercolony interactions (Suarez et al., 2002), thereby maintaining colony boundaries through avoidance of or aggression toward members of foreign conspecific colonies.

Invasive ant populations are often characterized by no or greatly reduced intraspecific aggression, which has radical consequences for their social organization. The lack of aggression toward non‐nestmates allows for the development of supercolonies, consisting of large polydomous colonies made of interconnected nests freely exchanging individuals (Eyer, McDowell, et al., 2018; Fournier et al., 2009; Holway et al., 2002). Supercolonies are associated with a collapse of colony boundaries resulting in a dramatic reduction in the level of relatedness among nestmates (Giraud et al., 2002; Passera & Keller, 1994; van der Hammen et al., 2002). This colony structure eliminates intraspecific competition and the cost of territorial fights. It therefore enables supercolonial invasive ants to reach extremely dense populations, which may become ecologically dominant and outcompete local species by monopolizing resources (Holway et al., 1998, 2002; Tsutsui et al., 2000).

Because nestmate recognition is at least partly influenced genetically (Walsh et al., 2020), the loss of a functioning nestmate recognition system in introduced populations is thought to stem from a lack of genetic differentiation between colonies, resulting in reduced diversity of the chemical recognition cues (Tsutsui et al., 2000, 2003). Species introductions are usually associated with reduced genetic diversity within invasive populations compared to native ones due to the founder effect (i.e., bottleneck). This overall reduction of diversity may also include loci influencing cuticular hydrocarbon production (Giraud et al., 2002; Lockey, 1991; Pirk et al., 2001). Ultimately, the loss of polymorphism at these loci could result in homogenized CHC templates between colonies, reducing the ability of workers to discriminate between nestmates and non‐nestmates (Giraud et al., 2002; Tsutsui et al., 2000, 2003; Vásquez et al., 2008). In addition, many invasive ant populations exhibit a polygyne social structure, whereby numerous reproductive queens are found within each nest. This social structure is suggested to enhance their invasion success by increasing colony growth and survival (Boomsma et al., 2014; Boulay et al., 2014), enabling polygyne colonies to produce and allocate a high number of workers to dominate resources (Tsutsui et al., 2000). However, this breeding system also increases the genetic diversity within colonies, including loci involved in nestmate recognition, due to the reproduction of several unrelated queens. The presence of multiple queens therefore broadens both the gestalt odor of the colony and the diversity of recognition cues accepted by their workers (Hölldobler & Wilson, 1977; Vander Meer & Morel, 1998), which may hamper proper nestmate recognition through the homogenization of the CHC template.

The dark rover ant, *Brachymyrmex patagonicus*, is native to South America and has recently become established in the southern USA (first report in 1976; Quirán et al., 2004; Wheeler & Wheeler, 1978). In 2007, this species has been reported in seven US states, but in less than a decade it has rapidly spread from coast to coast across 15 states and the Bahamas (Eyer et al., 2020; Guénard et al., 2012; Hill, 2017; MacGown et al., 2007; Martinez, 2010; Wheeler & Wheeler, 1978). More recently, this species has been reported in continental Asia (Guénard, 2018), Spain (Espadaler & Pradera, 2016), and Martinique (Carval et al., 2016). In addition, indoor occurrences of this species have been described in England, the Netherlands, and Japan, but it is uncertain whether outdoor populations can survive the harsh winters of these regions (Boer & Vierbergen, 2008; Terayama et al., 2014). *Brachymyrmex patagonicus* is a widespread and abundant pest infesting buildings and is increasingly becoming the subject of considerable indoor pest management efforts, although outdoors it does not draw much attention due to its tiny size (maximum length of workers 2.5 mm), and lack of biting or stinging (MacGown et al., 2007). Notably, in contrast with most invasive species, *B*. *patagonicus* does not form extensive supercolonies. Rather, each colony occupies a single nest, and the presence of a single or a few reproductive queens per colony enables significant relatedness among nestmates in many colonies (Eyer et al., 2020). A single queen, mated with up to four males, heads most of the colonies in four invasive populations in Texas, with only a fraction of the colonies (*ca*. 20%) being polygynous. This variation in the breeding system leads to a range of relatedness among nestmate workers between colonies, from genetically homogeneous colonies to more diverse ones (Eyer et al., 2020). Separate nests have been described in extremely close proximity (a few centimeters from each other), yet showed mutual tolerance, and populations were defined as extremely large, without an accurate estimation of colony density (MacGown et al., 2007). However, until these field observations and genetic findings are paired with behavioral and chemical evidence, several questions remain unanswered. It is still unclear how many *B. patagonicus* colonies cohabit a given locality and how foraging strategies allow them to partition resources. Similarly, the level of antagonism between different colonies and the maintenance of a functional nestmate recognition system remain unknown. Determining the genetic, chemical, or behavioral mechanisms that allow this tiny ant to maintain strict colony boundaries, and therefore to stand in sharp contrast with other invasive ant populations that have a supercolonial structure, will surely provide broader insights into ant invasions.

In this study, we investigated the interplay between genetic diversity, chemical recognition, and aggressive behaviors in a *multicolonial* invasive ant species. We first sought to investigate the density and spatial partitioning of *B. patagonicus* colonies in a population in its invasive range. Using genetic difference between sampled foraging trails, we assessed the number of colonies present in a given location and the number of foraging trails belonging to each colony. Second, we examined nestmate recognition in this species through aggression assays, determining whether workers belonging to different colonies recognize each other as nestmates or not. Finally, we investigated whether the amount of within‐colony CHC variation is associated with the genetic diversity within each colony, and whether the pairwise chemical and genetic differences between colonies determines the level of aggression displayed by workers.

## 2 MATERIALS & METHODS

### 2.1 Sampling

To estimate the population density of *B. patagonicus* in an urban site in its invasive range, workers from all active foraging trails were sampled on two residential properties in Bryan, TX, USA (properties *A* & *C*). All exterior walls and neighboring surfaces of each house were exhaustively searched for foraging trails up to 3–4 m away from the structure. Collection locations were mapped for each structure to estimate the distance between foraging trails. We did not collect full colonies, as *B. patagonicus* nests are often difficult to locate (MacGown et al., 2007). For each trail, collected individuals were directly stored in ethanol for subsequent genetic analyses to identify the number of unique colonies on each property and the number of active foraging trails from each colony. Each property was resampled (up to 20 trails) 1 month after the first sampling period to estimate colony stability or replacement over time. To assign trails to their colony of origin, four to six workers per trail were genotyped; while eight workers were genotyped for the eight colonies used for behavioral and chemical analyses (see below) to ensure robust estimates of genetic diversity.

### 2.2 Aggression assays

Behavioral assays were performed to determine whether workers exhibit aggression toward non‐nestmates and to correlate whether the aggression level between a pair of colonies varies with their genetic and chemical differences. Around 200 additional workers were collected from eight active trails (five on property *A* and three on property *C*; *A52*, *A56*, *A69*, *A70*, *A54*, *C65*, *C63*, and *C75*). The eight trails used for aggression assays were confirmed to belong to unique colony based on microsatellite analyses (see below), ensuring that behavioral assays were not biased by the use of unintentional pool of workers from distinct colonies. Aggression assays were performed within 1 day after collection. Assays were modified from Suarez et al. (1999). Two ants were placed into a 1‐cm‐diameter plastic arena, and their behavior was observed for 3 min under a microscope. The arena floor was covered with filter paper to prevent odor transfer between replicates, and the sides were coated with Fluon to prevent escape. Interactions between ants were scored using the following scale: 1 = ignoring, 2 = grooming or antennation, 3 = avoidance, 4 = aggression, 5 = fighting. Ignoring was defined as contact between ants with no further behaviors, whereas avoidance consisted of contact followed by retreat by one or both ants. Aggression included behaviors such as biting and spraying formic acid; an escalation of these behaviors with ants locking their mandibles onto one another and grappling constituted fighting. The aggression score for each assay was averaged across replicates for each colony pairing. We also measured aggression scores between nestmate workers. As a control, we isolated two groups of workers from the same colony for a 48‐hr period and re‐evaluated nestmate aggression between workers from each group. This control verifies that aggression between non‐nestmates does not result from a period of isolation under laboratory conditions after sampling. Overall, we conducted between four and seven replicates for each colony pairing (*N* = 64). In total, we had 145 aggression pairings between non‐nestmate workers, 40 between nestmates, and 10 between nestmates separated for 48 hr.

### 2.3 Genetic procedures and analyses

For each trail, the genomic DNA of four to eight workers was extracted following a modified Gentra Puregene extraction method (Gentra Systems, Inc.). Each worker was genotyped following PCR conditions and multiplexing arrangements at seven of the most polymorphic markers described in Eyer et al. (2020) (i.e., *Bpa7*, *Bpa8*, *Bpa9*, *Bpa13*, *Bpa14*, *Bpa16*, and *Bpa23*). PCRs were carried out on a Bio‐Rad thermocycler T100 (Bio‐Rad). PCR products were sized against the LIZ500 standard using an ABI 3500 capillary sequencer (Applied Biosystems). Allele calling was performed on Geneious v.9.1 (Kearse et al., 2012).

For each property, relatedness coefficients (*r*) between workers of each trail were estimated using COANCESTRY v.1.0 (Wang, 2011), following the algorithm described by Queller and Goodnight (1989). High relatedness values between workers sampled on a given trail would suggest that they belong to a single colony. Moderate relatedness values may denote the foraging trail of a single, yet more diverse, colony (e.g., polygyne), as Eyer et al. (2020) found the relatedness within polygyne colonies ranged from 0.16 to 0.64. In contrast, relatedness values close to zero would indicate that workers from different colonies forage on a common trail. Relatedness coefficients among trails were calculated separately for each property to avoid possibly inflated relatedness coefficients due to potential differences in genetic background between the two properties.

For each property, pairwise genotypic differentiation was tested between each pair of trails to determine whether they originated from the same colony using log‐likelihood *G* tests implemented in GENEPOP ON THE WEB (Rousset, 2008). A standard Bonferroni correction was performed to account for multiple comparisons. Genetic differentiation was determined for each pair of foraging trails by calculating pairwise *F*_ST_ using GENEPOP ON THE WEB (Rousset, 2008). The assignment of trails to colonies was visualized using the Bayesian clustering method implemented in STRUCTURE v.2.3.4 (Pritchard et al., 2000). For each property, simulations were run with *K* ranging from one to the number of trails sampled, with 20 replications for each number of *K*. The analyses were run using a combination of correlated‐allele frequencies and admixture modeling. Each run comprised a first step of a 50,000 burn‐in period and 100,000 iterations of the MCMC. The most likely number of genetic clusters was determined using the method of Evanno et al. (2005) implemented in Structure Harvester (Earl & vonHoldt, 2012). Finally, the clustering of the trails into colonies was also visualized by plotting individual genotypes on a principal component analysis (PCA) using the *adegenet* R package (Jombart, 2008). Based on the close genetic relationships among colony members, trails belonging to the same colony should cluster together, while trails from different colonies should segregate across the axes. Pairwise *F*_ST_ between colonies and an additional PCA analysis were performed on the eight colonies used for aggression assays and chemical differentiation.

### 2.4 Chemical procedures and analyses

The CHC profile of each colony used for aggression assays was assessed by analyzing four workers using GC‐MS. Each ant was immobilized for 1 min at −20°C. Due to the tiny size of the ants, extractions were performed in 5 µl of hexane for 10 min with regular gentle mixing directly within a 100‐μl insert in a 1.5‐ml GC autosampler vial. Ant bodies were removed with a pin. Samples were analyzed with an Agilent 7890B Gas chromatograph connected to an Agilent 5977B Mass Spectrometer with a splitless injector held at 250°C using ultrahigh‐purity helium as the carrier gas (0.75 ml/min constant flow rate). Injections (2 µl) were carried out using a 7683B Agilent autosampler into a HP‐5MS UI column (30 m × 0.250 mm internal diameter × 0.25 μm film thickness; Agilent). For each sample, the temperature of the column was held at 50°C for 1 min, increased to 310°C at 15°C/min, and held at 310°C for the last 10 min. Identification of compounds was completed through comparison of mass spectra with a mass spectral library (NIST17) and through comparison to an alkane standard. We only used compounds found in at least 10 individuals. We also only used compounds representing at least 1% of the relative proportion of cuticular hydrocarbons for each individual profile, in order to minimize contamination and artifact peaks. Overall, 20 chemical compounds were used in further analyses (Figure S1).

Principal component analysis (PCA) was used to plot the chemical differentiation within and between colonies. Colonies with different CHC profiles are expected to scatter across the axes of the PCA, whereas those with similar profiles are expected to cluster together. The level of CHC differentiation between each pair of colonies was calculated using the Euclidean distance between colony centroids. In addition, the level of CHC variation within each colony was calculated using the Mahalanobis distance between each pair of nestmate workers. In addition, random forest (RF) analysis was used to estimate the importance of each specific compound in explaining variation in aggression between each pair of colonies. A similar RF analysis was performed to estimate whether differences in specific compounds between a pair of colonies might be explained by their level of genetic differentiation (*F*_ST_).

### 2.5 Statistical analysis

We first investigated whether nestmate workers recognize each other and display aggressive behaviors toward non‐nestmates, by comparing aggression levels using Mann–Whitney tests. We investigated whether the level of aggression between each pair of colonies varies with their levels of genetic and chemical differentiation using linear regressions implemented in R core Team 2018. We also used linear regressions to test whether the level of CHC differentiation between colonies correlates with their level of genetic differentiation. In addition, we tested whether the level of CHC variation within a colony was negatively correlated with the level of genetic similarity within colonies (i.e., relatedness) using linear regressions. Random forest analyses were performed on R software using the *randomForest* package (Liaw & Wiener, 2002). We used the node purity estimates to interpret the importance of each compound in explaining aggression scores and genetic differentiation.

## 3 RESULTS

A total of 21 foraging trails were sampled on property *A* and 35 on property *C* during the initial investigation. An additional 19 and 20 trails from each property, respectively, were sampled 1 month after the initial investigation (Figure 2a). The final dataset thus includes 95 foraging trails sampled across the two properties over two sampling periods. As four to eight workers per trail were successfully genotyped (mean ± *SD* = 4.94 ± 1.20), the final dataset includes 427 workers genotyped at seven polymorphic microsatellites markers, with allele numbers ranging from 3 to 15 (mean ± *SD* = 8.86 ± 4.81).

**FIGURE 1:**
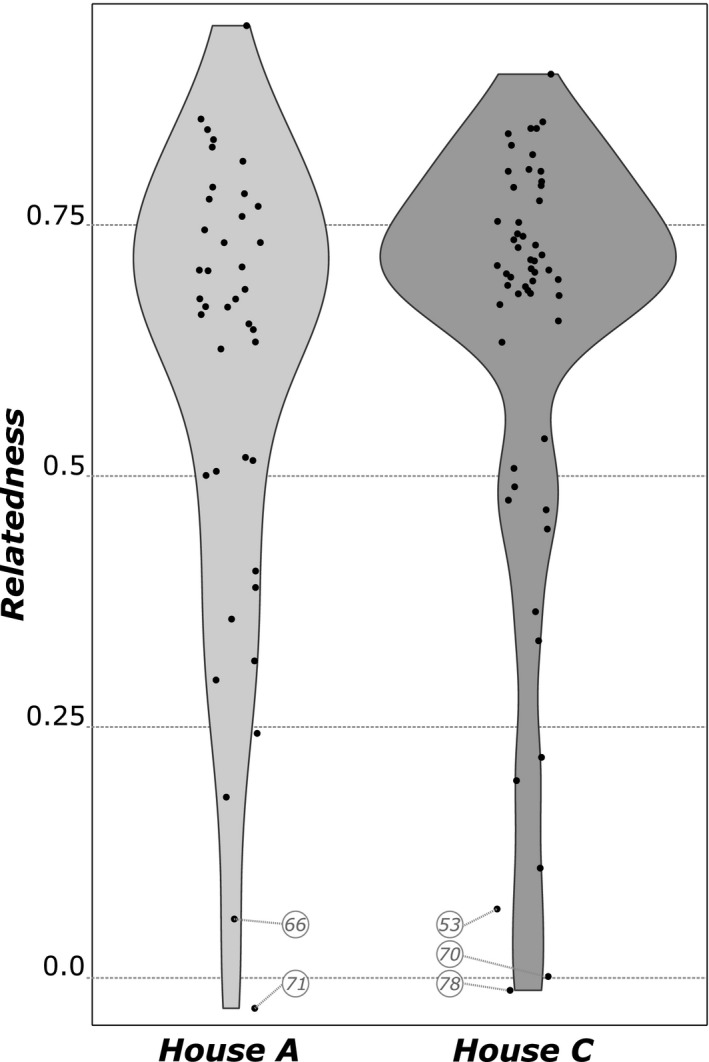
Violin plots of relatedness values between workers within each foraging trail for each of the two sampled properties. Each point represents the relatedness within a given trail. The trail identities are indicated for low‐relatedness trails (*R*_W‐W_ < 0.10)

**FIGURE 2:**
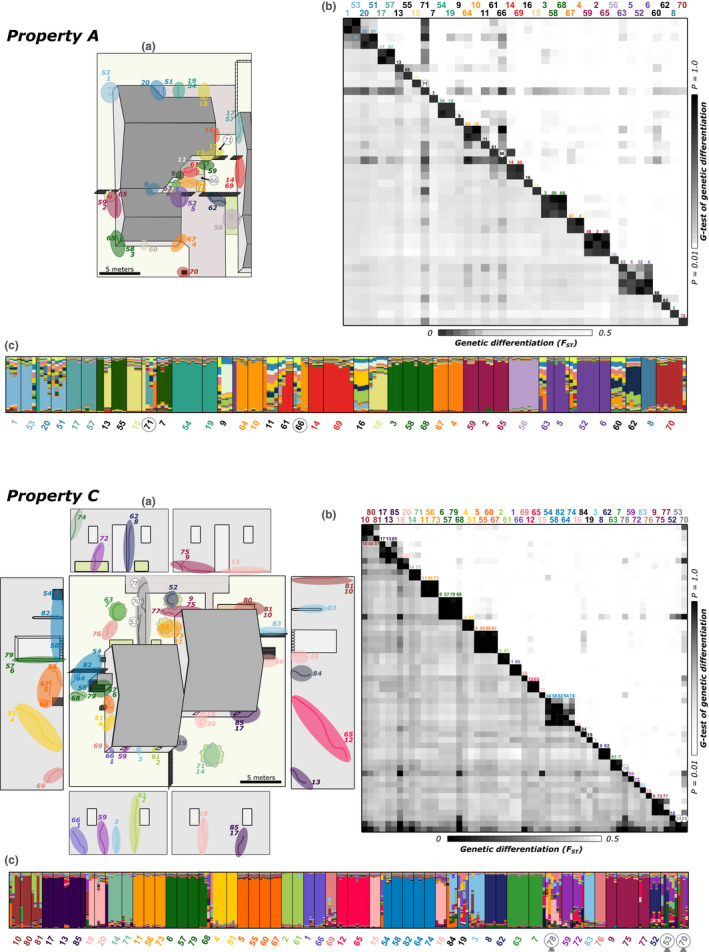
(a) Graphical representation of the foraging trails collected on each property. (b) Pairwise matrix of genetic differentiation for each study property. *F*_ST_ values between each pair of trails are provided below the diagonal, and values of the pairwise *G* tests of genetic differentiation are indicated above the diagonal. *F*_ST_ and *G* test values are colored using shades of gray. Low *F*_ST_ values and nonsignificant differences between pair of trails are depicted using dark colors, whereas high *F*_ST_ values and highly significant differences are indicated in light. (c) Bayesian clustering of individual workers into different genetic clusters (i.e., colony). Each genetic group is characterized by a color, and each individual is represented by a vertical bar according to its probability of belonging to each group. Arrows indicate low‐relatedness trails

Overall, workers from most trails likely belong to the same colony, as relatedness between workers from a given trail was relatively high (*R*_w‐w_ ± *SD* = 0.62 ± 0.22; Figure 1). This therefore suggests that workers from different colonies do not share foraging trails. Overall, only five trails showed a low level of relatedness (*R*_w‐w_ < 0.10), which could potentially signify that workers from different trails belonging to different colonies were unintentionally pooled. Within each property, pairwise genetic differentiation tests confirmed that most of the trails sampled could be genetically assigned to a distinct colony (Figure 2b; detailed results of pairwise *F*_ST_ and genotypic differentiation between trails are provided in Figure S2). On property *A*, 24 genetically different colonies were inferred across the 40 trails sampled, with trails belonging to the same colony being those resampled during the second sampling period or trails spatially close to each other. A similar pattern was observed on property *C*, as 29 genetically different colonies were found across the 55 trails sampled, with distinct trails from a given colony always adjacent, or were resampled trails. Clustering of trails into different colonies can also be visualized using structure analyses, clustering properties *A* and *C* into *K* = 21 and 18 genetic clusters, respectively (Figure 2c), with most colonies forming unique genetic clusters. Unsurprisingly, discrepancies between structure assignment and *G* test results mostly stem from the difficulty of grouping workers from highly diverse trails (i.e., low relatedness) into a single genetic cluster. Genetic clustering of the trails into the different colonies was also evident using principal component analysis, as the different colonies scattered along the axes, while different trails from the same colonies cluster together (Figure 2d).

Overall, across the two properties, 39 colonies were associated with a single trail, 11 colonies had two trails, two colonies had three trails, and a single colony occupied four trails (Figure 2a). Interestingly, no overlap was found between trails of different colonies, suggesting that colonies of this species maintain distinct territories. Similarly, among the 39 trails sampled a month apart, 23 trails could be assigned to previously collected colonies and occupied the exact same locations (10 on property *A*, 13 on property *C*). Two trails were assigned to colonies previously sampled, but were collected a couple of meters away from the first sampling (one on each property), while the 14 others represent 12 new colonies (8 on property *A*, 6 on property *C*). On property *A*, the 24 colonies found across the 78.4 linear meters investigated represented a different colony found every 3.2 m, while a different colony was found every 2.5 m on property *C* (29 colonies in 71.6 linear meters).

### 3.1 Aggression assays

**FIGURE 3:**
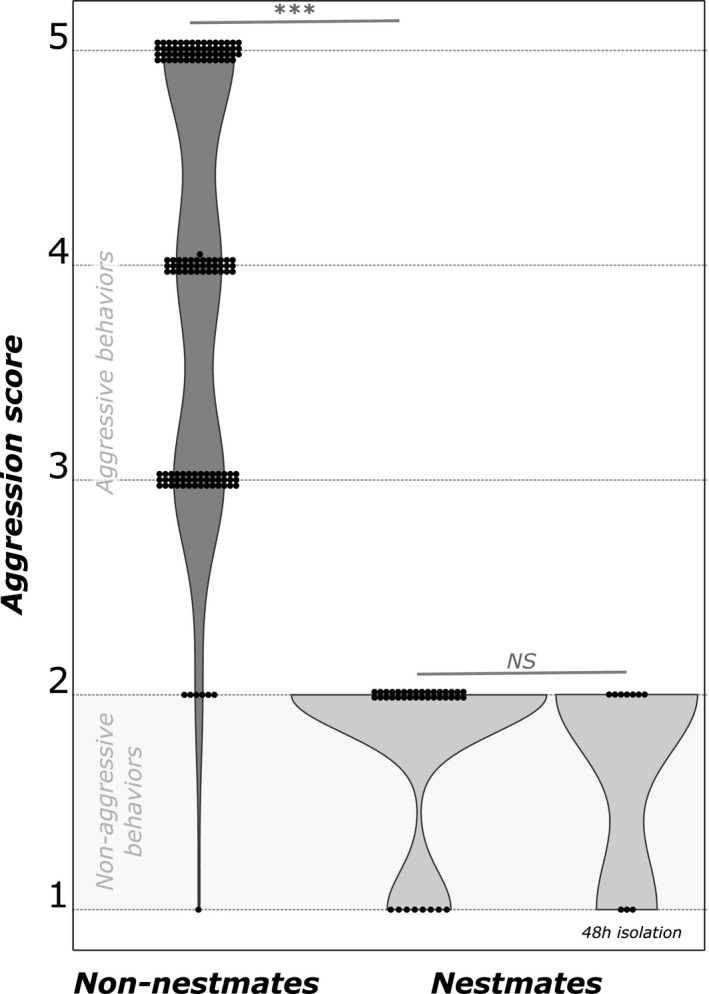
Violin plots of aggression level between nestmate or non‐nestmate workers of *B. patagonicus*. Each point represents the aggression result of a paring between two workers. Gray zone indicates nonaggressive behaviors (a score from 0 to 2)

Aggressive behaviors were observed in almost all behavioral assays of *B. patagonicus* between non‐nestmates workers, as only seven (4.8%) of the 145 replicates exhibited an aggression score of 1 or 2 (Figure 3). As a result, the average aggression score between workers belonging to two distinct colonies ranged from 2.8 to 5.0 (mean ± *SD* = 4.01 ± 0.51). In comparison, aggressive behaviors were never observed in assays involving nestmates workers, resulting in an average score of 1.8 (± *SD* = 0.15). Consequently, *B. patagonicus* workers were significantly more aggressive toward non‐nestmates than toward nestmate workers (Mann–Whitney test, *p* < 0.001) and they showed no aggression toward nestmates after the 48‐hr period of separation (Mann–Whitney test, *p* = 0.51).

### 3.2 Interplay between chemical profiles, genetic composition, and behavior

**FIGURE 4:**
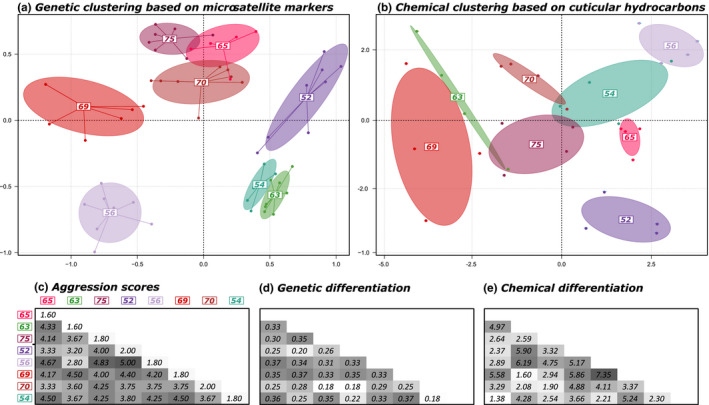
Clustering of the eight foraging trails used for behavioral and chemical assays based on principal component analyses of microsatellite genotypes (a) and chemical profiles (b). Matrices of aggression scores (c), pairwise genetic differentiation *F*_ST_ (d), and chemical differentiation (e) between each pair of colonies

The eight trails used for the aggression assays belong to genetically distinct colonies (Figures 2 and 4a). Cuticular hydrocarbons were extracted and analyzed for four workers for each of those colonies. Different colonies were found to be chemically differentiated, as the distinct colonies scattered along the axes of the principal component analysis (Figure 4b), while most of the individuals within a given colony cluster together (the pairwise distances between each pair of individuals are given in Figure S4). As a result, the average pairwise distance between individuals from distinct colonies (mean ± *SD* = 6.40 ± 1.25) was significantly higher than the average distance between nestmates (mean ± *SD* = 3.82 ± 0.87; ranges from 2.64 to 4.81) (*p* < 0.001; Figure S4).

**FIGURE 5:**
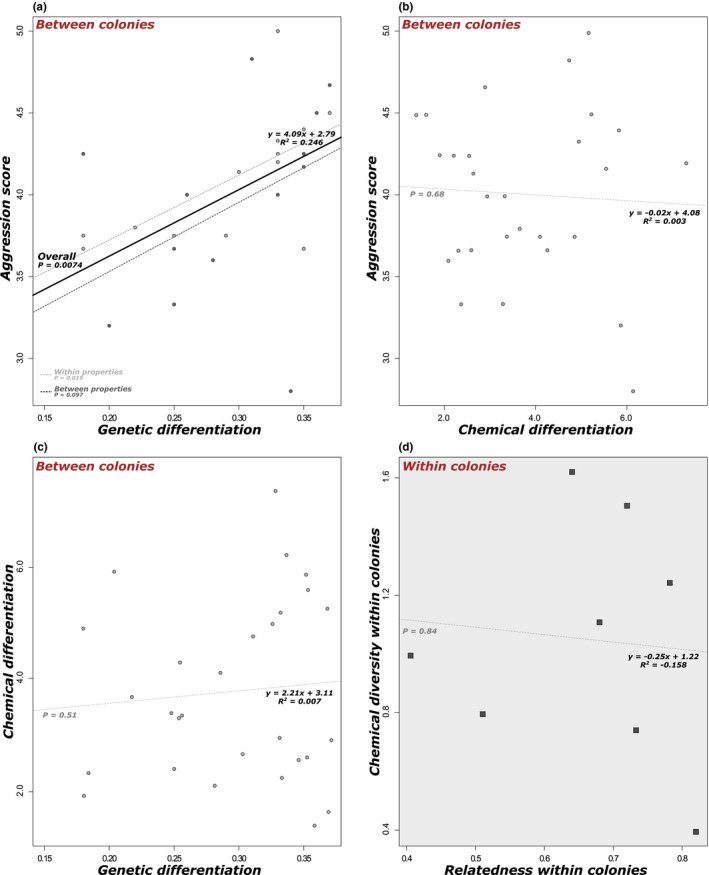
Linear regressions between values of pairwise genetic differentiation, chemical differentiation, and aggression scores between each pair of colonies

We found a significant correlation between the genetic differentiation between colonies (*F*_ST_) and their level of aggression (*p* = 0.0074; Figures 4c,d and 5a). However, we did not find any significant relationship between the degree of chemical differentiation between colonies (i.e., Euclidean distance between centroids) and their level of aggression (*p* = 0.68; Figures 4c,e and 5b). Similarly, no significant relationship was found between the degree of genetic differentiation between colonies and their level of chemical differentiation (*p* = 0.51; Figures 4d,e and 5c). Within colonies, the level of chemical diversity (i.e., Mahalanobis distance) was not significantly correlated with the relatedness among nestmates (i.e., level of genetic homogeneity within colonies) (*p* = 0.59; Figure 5d). When assessing each compound separately using random forest analyses, we found that pairwise difference in a single compound cannot fully account for the level of aggression observed between colonies (Figure S5). A positive, yet not significant, correlation between aggression and differences in production was found for two compounds. The level of aggression between a pair of colonies increased with their differences in C23 Alkene (*p* = 0.073) and C36 Alkane (*p* = 0.068; Figure S5).

## 4 DISCUSSION

Our study provides several insights into the colony structure of *Brachymyrmex patagonicus*, a multicolonial invasive ant. Our results revealed that this species can reach high densities, with a different colony found every 2.5 m, while still maintaining strict colony boundaries through aggressive behaviors. Our results also revealed that each colony uses one or a few foraging trails, which consistently remain in the same location. This stable foraging strategy may lower intraspecific competition and favor high density through efficient resource partitioning and an avoidance of rival colonies. The antagonism toward non‐nestmates and the chemical differentiation between colonies indicate that this invasive species maintains a functional nestmate recognition system, with the level of aggression between two colonies being associated with their level of genetic differentiation. Together, these findings suggest that the loss of genetic diversity during this species' introduction was not enough to lower the diversity of loci involved in nestmate recognition to the point that it disabled worker discrimination. This may suggest that either the species did not experience a strong bottleneck during its introduction, potentially through multiple introductions, or that the recognition system of this species is resilient to a loss of genetic diversity.

The close proximity of neighboring colonies found in this study suggests that there are abundant resources available or that different colonies are able to adequately partition them (Levings & Franks, 1982; Ryti & Case , 1986). Similar to other urban pest ant species, *B. patagonicus* is a generalist and has been reported foraging on various carbohydrate‐rich and proteinaceous food items, depending on availability or seasonality (Edwards & Abraham, 1990; Keefer, 2016). The landscaping around homes therefore provides this generalist species ample food resource (i.e., extrafloral nectar, honeydew, and dead insects) and numerous nesting sites (MacGown et al., 2007; Navarrete et al., 2013). Most trails of *B. patagonicus* were found at the same location 1 month later suggesting that food resources were fairly stable over this time period. In contrast to solitary foraging, consistent trail foraging over time may favor resource partitioning and territoriality, limiting encounters with non‐nestmate workers (Bernstein, 1975; Hölldobler, 1976). The maintenance of territorial behavior requires that the benefits of territoriality outweigh the costs of territorial defense. Territorial costs can be reduced by limiting antagonism toward frequently encountered workers from neighboring colonies (i.e., dear enemy effect). However, the opposite is also found in some species, whereby aggression is higher toward neighbors than random strangers (i.e., nasty neighbor effect), such as in *Oecophylla smaragdina* (Newey et al., 2010) and *Pristomyrmex pungens* (Sanada‐Morimura et al., 2003). In some species, workers indiscriminately fight workers from neighboring and non‐neighboring colonies, such as in *Camponotus cruentatus* (Boulay et al., 2007) and *Iridomyrmex purpureus* (Thomas et al., 1999). Overall, aggression toward non‐nestmates leads colonies to antagonistically avoid each other and overdisperse across the landscape. Consequently, intraspecific competition increases with colony density in territorial ants. In *B. patagonicus*, the ant's ability to forage on different resources, the overdispersion of the colonies, as well as the tiny size of individuals and the small size of the colonies (i.e., probably not requiring large amounts of food; MacGown et al., 2007) may allow populations to sustain high density of aggressive colonies without depleting resources, such as extrafloral nectar and honeydew (Lanan, 2014; Traniello, 1989).

The colony density of *B. patagonicus* observed in this study corresponds to 400 to 800 colonies per hectare. Unfortunately, no information on colony density is available for this species in its native range. The monogyne form of the fire ant *Solenopsis invicta*, one of the other monogyne invasive ant species, exhibits a density of 295 mounds/ha in its invasive range in Texas, which is 3–4 times higher than in its native range (Porter et al., 1991). In contrast, the polygyne form of this ant occurs at much higher densities (680 mounds/ha; Porter et al., 1991) in the invasive range, despite the fact that the monogyne and polygyne forms seem to experience similar levels of intraspecific competition (Kjeldgaard et al., 2020). Notably, the numerous *B. patagonicus* colonies identified in this study were found in spite of the presence of fire ant colonies, which suggests that these two invasive species forage on different resources and do not mutually exclude each other.

Most trails analyzed in this study exhibited a relatively high level of relatedness, indicating the occurrence of a single reproductive queen in most colonies. These findings confirm previous results that a majority of invasive colonies of *B. patagonicus* are monogyne (Eyer et al., 2020). This study also highlights the need for multiple tools to efficiently infer the colony boundaries in socially complex species (Ellis et al., 2017; Eyer et al., 2017; Reiner Brodetzki & Hefetz, 2018). Although the colony structure of this species in its native range in South America is unknown, it likely exhibits a similar multicolonial and monogyne structure. Interestingly, a growing number of ant species are reported as successful invaders despite exhibiting large supercolonies (Eyer & Vargo, 2021). This includes other multicolonial species, such as *S. invicta* (Kjeldgaard et al., 2020) or *Tetramoriun immigrans* (Zhang et al., 2019). It also includes species where invasive colonies are large and made of several interconnected nests, but their size does not exceed 1 km in length, such as *Myrmica rubra* (Chen et al., 2018), *Brachyponera chinensis* (Eyer, Matsuura, et al., 2018), and *Technomyrmex albipes* (Yamauchi et al., 1991). Most invasive supercolonial species often exhibit a lower degree polygyny and supercolonies of smaller size in their native range (Errard et al., 2005). These pre‐existing characteristics have been suggested to represent a stepping‐stone to supercoloniality after experiencing a glitch in their nestmate recognition system through a loss of genetic diversity (Errard et al., 2005). Our results may provide partial support for this hypothesis, as the potential loss of diversity alone has not resulted in a malfunctioning recognition system and the formation of supercolonial invasive population in *B. patagonicus*, presumably because native populations do not have the requisite pre‐existing flexibility in their social organization.

In many invasive social insects, a loss of genetic diversity in the invasive range leads to a decrease in genetic differentiation among invasive colonies. This results in a homogenization of both the gestalt odor of distinct colonies and the recognition threshold accepted by workers, which are more likely to accept non‐nestmates with comparable chemical profiles (Vander Meer & Morel, 1998). Together, these features may generate a positive feedback loop, whereby a reduction in recognition ability favors the adoption of unrelated queens, hence further increasing the genetic diversity within colonies and decreasing the differentiation between colonies. This positive feedback has been suggested to enable the development of supercolonies in bottlenecked invasive populations (Giraud et al., 2002; Tsutsui et al., 2000). For example, in the invasive wasp *Vespula pensylvatica*, low nestmate discrimination allows the reintroduction of new queens, resulting in large polygyne colonies (Hanna et al., 2014; Loope et al., 2018). Similarly, Argentine ant workers more readily adopt foreign queens when their profiles are similar to those of nestmate queens (Vásquez et al., 2008). As the large supercolonies in California, Hawaii, Australia, and Spain experienced a common loss of genetic and chemical variation compared to the native range, they share similar genetic and chemical profiles (Brandt et al., 2009) and are not aggressive to each other despite their worldwide distribution (Van Wilgenburg et al., 2010).

Currently, we do not know whether invasive populations of *B. patagonicus* (Eyer et al., 2020) experienced a genetic bottleneck, as no data are yet available on the genetic diversity in the native range. The maintenance of colony boundaries and pronounced genetic and chemical differentiation between colonies may possibly be explained by a limited loss of genetic diversity in its invasive range, preserving the ability of workers to discriminate non‐nestmates thereby maintaining strict colony boundaries. Relatively high levels of genetic and chemical diversity in the introduced range may occur through multiple introductions, whereby different introductions originating from distinct native populations increase the amount of genetic and chemical variation brought to the introduced range (Dlugosch & Parker, 2008). For example, the European population of the Argentine ant can be divided into two genetically, chemically, and behaviorally distinct supercolonies, arising from two separate introduction events from different localities in the native range (Brandt et al., 2009; Mothapo & Wossler, 2011; Van Wilgenburg et al., 2010). Similarly, the two supercolonies of the Western Cape (South African) population of this species also originated from distinct native localities. The tiny size of *B. patagonicus* may potentially increase its chances of being transported, and therefore the chance of multiple introductions into the USA (MacGown et al., 2007), thus reducing the bottleneck effect. This invasive species has recently become established in many places around the world (Espadaler & Pradera, 2016; Guénard, 2018). Future studies may focus on whether other introduced populations of *B. patagonicus* exhibit the same behavioral, genetic, and chemical patterns. We therefore cannot rule out that other invasive populations of *B. patagonicus* exhibit a supercolonial structure as distinct invasive populations likely experienced different invasion histories, affecting the amount of genetic and chemical diversity present in each of them (Bertelsmeier et al., 2018; Blumenfeld et al., 2021).

Although the chemical distance between colonies is associated with their overall level of genetic differentiation in some ant species (e.g., Reiner Brodetzki & Hefetz, 2018), this association is not found in other ant species (e.g., Frizzi et al., 2015). In invasive social insects, the “genetic cleansing” hypothesis suggests that recognition ability is lost when polymorphism at the recognition loci is reduced (Giraud et al., 2002). As this hypothesis assumes different selective pressures acting on recognition loci than the rest of the genome, the lack of recognition is therefore not always associated with an overall reduction of genetic diversity observed at neutral markers (i.e., microsatellites). According to this hypothesis, the loss of recognition ability in introduced populations of the Argentine ant occurred despite a limited loss of genetic diversity at neutral markers (Giraud et al., 2002). This possibility is supported by the finding that nestmate recognition may not be based on the overall CHC profiles, but may rely upon few specific chemical compounds used as recognition cues (Martin et al., 2008a, 2008b,2008a, 2008b). In addition to nestmate recognition, CHC profiles encode other recognition signals, such as species, fertility, and task information (Denis et al., 2006) and change over time across different environments. The selection acting upon compounds used as nestmate recognition cues may differ from those shaping the rest of the cuticular hydrocarbons. The analysis of overall CHC profiles may therefore mask and dilute the influence of compounds used in nestmate recognition. Consequently, accurately assessing the impact of selection/introduction on recognition cue diversity requires teasing apart the role of each compound in nestmate recognition (Helanterä et al., 2011; Martin et al., 2008a, 2008b,2008a, 2008b). In this study, we did find evidence of distinct CHC profiles among different colonies; however, we did not find any correlation between genetic and chemical differentiation, which may suggest that other factors influence variation in chemical profiles, with only a fraction of the variation having an impact on nestmate recognition and/or having a genetic basis. Similarly, we found an absence of correlation between aggression and chemical differentiation while analyzing the overall profile of individuals of *B. patagonicus*. This suggests that even minor differences in CHC profiles among *B. patagonicus* colonies could be sufficient for workers to distinguish. While investigating the identity of chemical compounds underlying nestmate recognition in this species, we found that the level of aggression between a pair of colonies increases with their differences in C23 Alkene and C36 Alkane, but the association was not significant (Figure S5). This suggests that nestmate recognition does not rely on a single compound in *B. patagonicus*. If a large number of compounds encode recognition cues, the nestmate recognition system may be more resistant *chemical cleansing* during introduction, because it is less likely that they would all be affected. For example, *Formica exsecta* has a very simple CHC profile; the chemical profile of the Z9‐alkenes was the only significant predictor of aggression between colonies, leading to frequent errors of recognition (Martin et al., 2012). In *L. humile* and *W. auropunctata,* the cuticular profiles of introduced populations are less diverse relative to the native range, but supercolonial populations still possess a large number of methyl‐branched hydrocarbons used as recognition cues (Brandt et al., 2009; Errard et al., 2005). Finally, similar to selection on chemical production, selection may also act upon chemosensory systems enabling their detection. Genomic analyses reveal that ants have evolved a large number of odorant receptor genes (Nygaard et al., 2011; Smith et al., 2011; Wurm et al., 2011). Therefore, the formation of supercolonial populations may not simply result from a loss of overall genetic or chemical diversity, but may result from a complex interplay between their production, their roles as recognition cues, and the mechanisms enabling their detection.

## CONFLICT OF INTEREST

None declared.

## AUTHOR CONTRIBUTION

**Pierre‐André Eyer:** Conceptualization (lead); Data curation (lead); Formal analysis (lead); Investigation (lead); Methodology (lead); Writing‐original draft (lead); Writing‐review & editing (lead). **Phillip Shults:** Conceptualization (supporting); Data curation (supporting); Funding acquisition (lead); Investigation (supporting); Methodology (supporting); Writing‐original draft (supporting). **Madeleine R. Chura:** Data curation (equal); Investigation (equal); Methodology (equal). **Megan N. Moran:** Data curation (equal); Investigation (equal); Methodology (equal). **Morgan N. Thompson:** Data curation (supporting); Investigation (supporting); Methodology (supporting). **Anjel M. Helms:** Data curation (equal); Funding acquisition (equal); Investigation (equal); Methodology (equal); Writing‐original draft (equal). **Raj K. Saran:** Conceptualization (equal); Funding acquisition (equal); Methodology (equal). **Edward Vargo:** Conceptualization (equal); Funding acquisition (equal); Methodology (equal); Project administration (equal); Supervision (equal); Writing‐original draft (equal).

## Supporting information

Figure S1

Figure S2

Figure S3

Figure S4

Figure S5

Table S1

## Data Availability

The data reported in this study are deposited in Dryad database (https://doi.org/10.5061/dryad.h70rxwdhx).
