# Supplementary figures and images for: Divide and conquer: Multicolonial structure, nestmate recognition, and antagonistic behaviors in dense populations of the invasive ant Brachymyrmex patagonicus
Source: Ecol Evol. 2021 Mar 18;11(9):4874–86. doi: 10.1002/ece3.7396 (PMC8093738; doi:10.1002/ece3.7396)

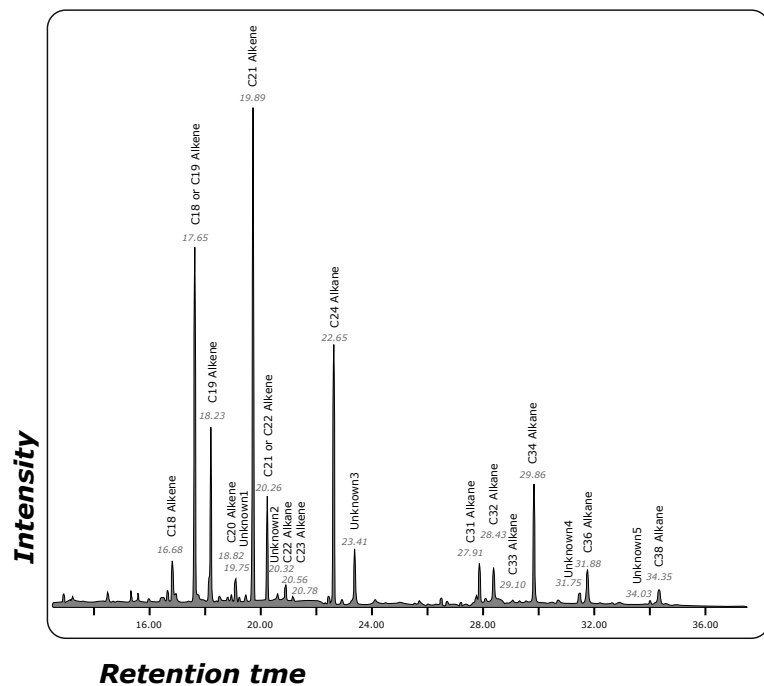

Supplement: Supplementary file 1 — Figure S1 [file ECE3-11-4874-s004.pdf]

## PCA clustering of foraging trails

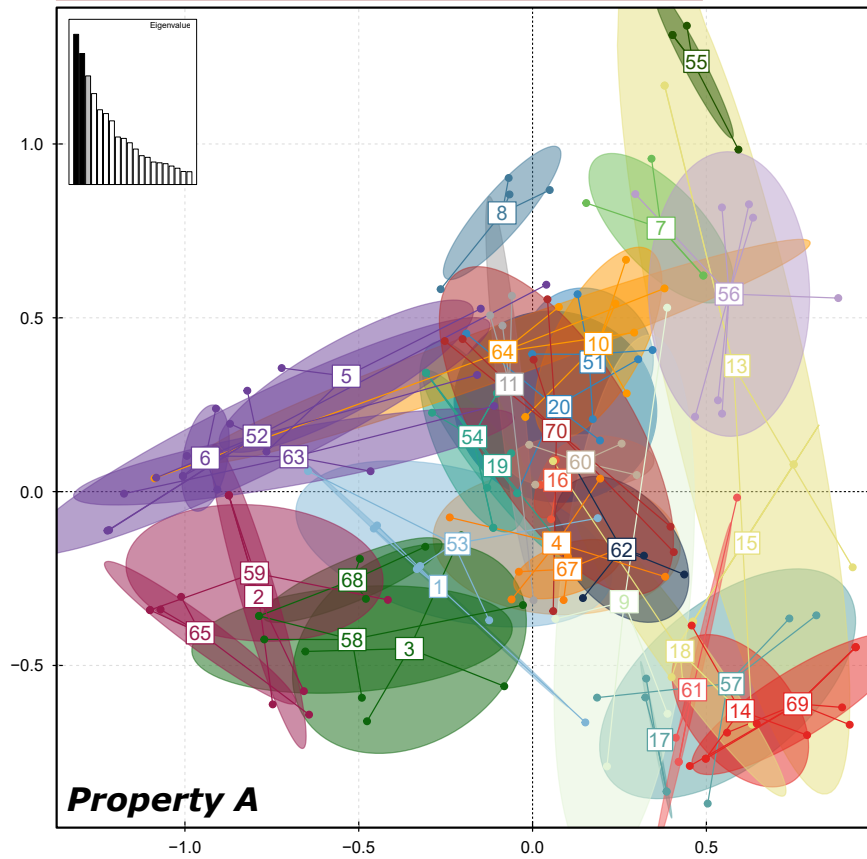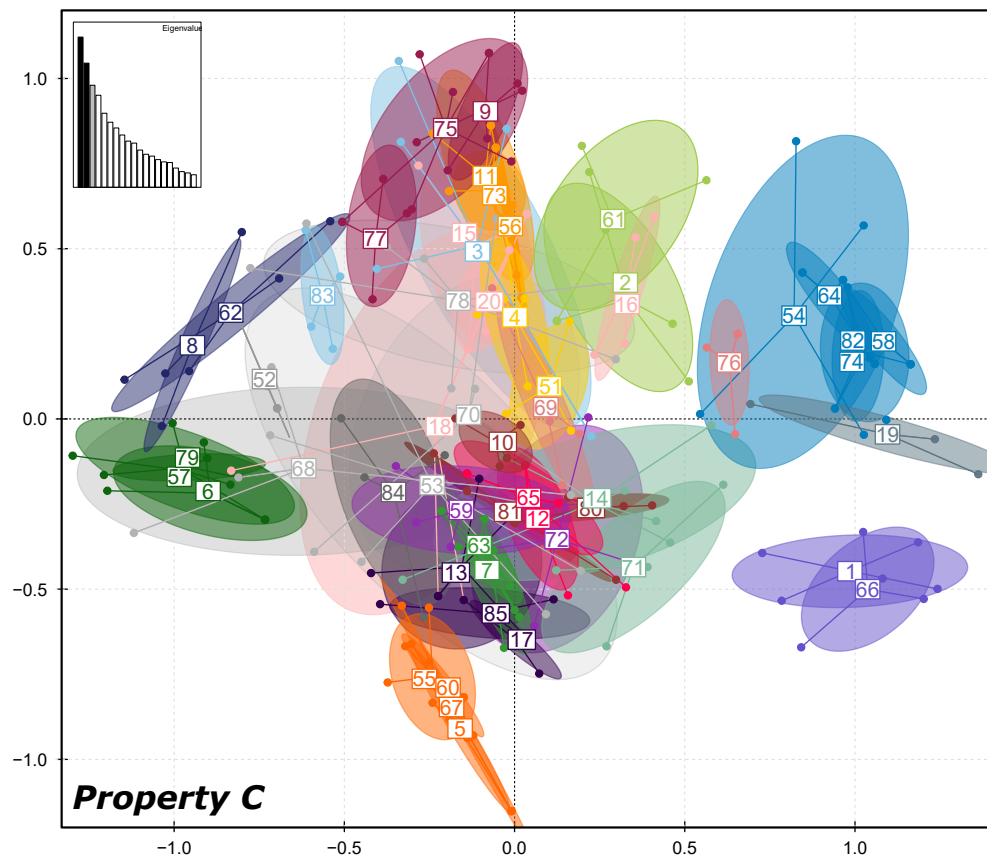

Supplement: Supplementary file 3 — Figure S3 [file ECE3-11-4874-s001.pdf]
