# Supplementary material for: Divide and conquer: Multicolonial structure, nestmate recognition, and antagonistic behaviors in dense populations of the invasive ant Brachymyrmex patagonicus
Source: Ecol Evol. 2021 Mar 18;11(9):4874–86. doi: 10.1002/ece3.7396 (PMC8093738; doi:10.1002/ece3.7396)
Supplement: Supplementary file 2 — Figure S2 [file ECE3-11-4874-s005.pdf]

	A_01	A_53	A_20	A_51	A_17	A_57	A_13	A_55	A_15	A_71	A_07	A_54	A_19	A_09	A_64	A_10	A_11	A_61	A_66	A_14	A_69	A_16	A_18	A_03	A_58	A_68	A_67	A_04	A_59	A_02	A_65	A_56	A_63	A_05	A_52	A_06	A_60	A_62	A_08	A_70
A_01	1.000	1.000	0.081	0.037	0.001	0.002	0.000	0.000	0.000	0.699	0.000	0.000	0.001	0.005	0.001	0.000	0.004	0.136	0.014	0.008	0.000	0.008	0.000	0.008	0.006	0.002	0.001	0.000	0.007	0.001	0.003	0.000	0.018	0.001	0.000	0.000	0.002	0.001	0.009	0.000
A_53	-0.0378	1.000	0.176	0.034	0.002	0.001	0.000	0.000	0.000	0.569	0.000	0.000	0.000	0.001	0.003	0.000	0.009	0.020	0.020	0.003	0.000	0.020	0.000	0.003	0.001	0.001	0.000	0.000	0.005	0.001	0.001	0.000	0.015	0.002	0.000	0.000	0.003	0.002	0.003	0.000
A_20	0.0783	0.0696	1.000	0.957	0.002	0.016	0.010	0.000	0.000	0.829	0.000	0.000	0.166	0.031	0.041	0.001	0.123	0.065	0.400	0.004	0.000	0.004	0.000	0.002	0.001	0.001	0.000	0.000	0.001	0.000	0.001	0.000	0.034	0.006	0.000	0.000	0.017	0.003	0.017	0.000
A_51	0.1193	0.1012	-0.0152	1.000	0.001	0.003	0.001	0.000	0.000	0.113	0.000	0.000	0.001	0.003	0.011	0.000	0.002	0.006	0.229	0.000	0.000	0.002	0.000	0.000	0.000	0.000	0.000	0.000	0.001	0.002	0.000	0.000	0.002	0.001	0.000	0.000	0.003	0.002	0.000	0.000
A_17	0.2929	0.2821	0.2626	0.3137	1.000	1.000	0.001	0.000	0.000	0.417	0.000	0.000	0.002	0.003	0.000	0.000	0.000	0.019	0.013	0.005	0.000	0.000	0.000	0.043	0.013	0.004	0.000	0.000	0.003	0.001	0.000	0.000	0.001	0.002	0.000	0.002	0.001	0.004	0.000	0.000
A_57	0.311	0.2947	0.2477	0.298	-0.0345	1.000	0.001	0.000	0.000	0.435	0.000	0.000	0.001	0.011	0.001	0.000	0.001	0.008	0.120	0.003	0.000	0.000	0.000	0.025	0.005	0.003	0.000	0.000	0.002	0.001	0.000	0.000	0.001	0.007	0.000	0.004	0.005	0.005	0.000	0.000
A_13	0.2704	0.2387	0.1809	0.2162	0.2641	0.2539	1.000	0.176	0.111	0.156	0.020	0.000	0.007	0.027	0.053	0.015	0.023	0.011	0.039	0.009	0.000	0.000	0.000	0.001	0.002	0.001	0.001	0.001	0.000	0.000	0.000	0.000	0.001	0.001	0.000	0.000	0.047	0.000	0.000	0.000
A_55	0.4466	0.404	0.3179	0.322	0.4761	0.4607	0.0891	1.000	0.000	0.000	0.000	0.000	0.000	0.000	0.001	0.001	0.000	0.000	0.000	0.000	0.000	0.000	0.000	0.000	0.000	0.000	0.000	0.000	0.000	0.000	0.000	0.000	0.000	0.000	0.000	0.000	0.001	0.000	0.000	0.000
A_15	0.372	0.3613	0.3382	0.3897	0.3731	0.3781	0.1257	0.4	1.000	0.027	0.000	0.000	0.002	0.031	0.002	0.001	0.003	0.001	0.007	0.000	0.000	0.000	0.001	0.001	0.004	0.000	0.000	0.001	0.000	0.000	0.000	0.000	0.000	0.000	0.000	0.000	0.007	0.000	0.000	0.000
A_71	0.0595	0.056	0.0343	0.1041	0.0582	0.0517	0.0868	0.3352	0.1746	1.000	0.002	0.000	0.069	0.316	0.162	0.003	0.364	0.118	0.740	0.135	0.000	0.040	0.258	0.215	0.162	0.060	0.032	0.019	0.088	0.057	0.047	0.000	0.056	0.013	0.000	0.009	0.147	0.094	0.079	0.057
A_07	0.4336	0.387	0.3157	0.3229	0.4783	0.4518	0.1795	0.2405	0.4229	0.318	1.000	0.000	0.000	0.002	0.012	0.010	0.000	0.003	0.002	0.001	0.000	0.000	0.000	0.000	0.000	0.000	0.000	0.000	0.000	0.000	0.000	0.000	0.000	0.001	0.000	0.000	0.002	0.000	0.001	0.000
A_54	0.3302	0.3147	0.1825	0.3203	0.3762	0.3758	0.3169	0.4216	0.4178	0.2103	0.4423	1.000	0.790	0.000	0.000	0.000	0.000	0.000	0.000	0.000	0.000	0.000	0.000	0.000	0.000	0.000	0.000	0.000	0.000	0.000	0.000	0.000	0.000	0.000	0.000	0.000	0.000	0.000	0.000	0.000
A_19	0.3308	0.3133	0.172	0.3308	0.3778	0.3711	0.2982	0.4427	0.4229	0.1647	0.4757	-0.0111	1.000	0.013	0.006	0.001	0.071	0.028	0.037	0.004	0.000	0.001	0.000	0.011	0.008	0.002	0.000	0.001	0.001	0.001	0.001	0.000	0.001	0.003	0.000	0.000	0.025	0.017	0.004	0.000
A_09	0.2789	0.2814	0.2466	0.3072	0.2566	0.2693	0.137	0.3354	0.2204	0.1315	0.3333	0.3275	0.3144	1.000	0.003	0.000	0.005	0.064	0.128	0.011	0.000	0.001	0.000	0.010	0.012	0.004	0.001	0.000	0.001	0.002	0.001	0.000	0.003	0.003	0.000	0.002	0.012	0.000	0.001	0.000
A_64	0.2313	0.2236	0.1615	0.1862	0.2989	0.2856	0.1906	0.2646	0.3079	0.138	0.2851	0.271	0.2532	0.205	1.000	0.997	0.178	0.017	0.977	0.003	0.000	0.001	0.000	0.009	0.004	0.001	0.012	0.006	0.005	0.004	0.000	0.000	0.134	0.005	0.000	0.018	0.130	0.002	0.002	0.000
A_10	0.3498	0.3373	0.2562	0.2883	0.3623	0.3333	0.2263	0.3034	0.36	0.2285	0.331	0.3335	0.3287	0.2654	-0.0106	1.000	0.039	0.005	0.390	0.000	0.000	0.001	0.000	0.000	0.000	0.000	0.000	0.000	0.000	0.000	0.000	0.000	0.000	0.000	0.000	0.000	0.060	0.000	0.001	0.000
A_11	0.216	0.1916	0.1229	0.2187	0.3056	0.2941	0.1967	0.3113	0.3061	0.081	0.3419	0.1841	0.1535	0.2579	0.0709	0.1472	1.000	0.008	0.301	0.003	0.000	0.010	0.000	0.010	0.003	0.001	0.009	0.019	0.005	0.003	0.000	0.000	0.037	0.008	0.000	0.000	0.022	0.018	0.003	0.000
A_61	0.2448	0.2522	0.2105	0.2892	0.251	0.2318	0.2473	0.4444	0.3483	0.088	0.4402	0.3172	0.3069	0.1795	0.1955	0.2482	0.2355	1.000	0.303	0.723	0.006	0.008	0.001	0.029	0.019	0.002	0.035	0.002	0.001	0.000	0.000	0.000	0.001	0.001	0.000	0.000	0.012	0.010	0.001	0.000
A_66	0.1375	0.1351	0.0533	0.083	0.1502	0.1176	0.1313	0.2505	0.24	0.0197	0.2716	0.2363	0.1915	0.1398	-0.0093	0.049	0.0631	0.0462	1.000	0.019	0.000	0.001	0.000	0.007	0.007	0.003	0.033	0.007	0.002	0.001	0.001	0.000	0.027	0.021	0.000	0.036	0.266	0.020	0.003	0.000
A_14	0.2457	0.23	0.2107	0.2774	0.2301	0.2325	0.1839	0.4269	0.3062	0.0782	0.3771	0.3378	0.3411	0.1918	0.2569	0.318	0.2691	0.0964	0.1223	1.000	0.851	0.001	0.000	0.007	0.002	0.000	0.002	0.000	0.000	0.000	0.000	0.000	0.000	0.000	0.000	0.000	0.001	0.003	0.000	0.000
A_69	0.2658	0.2647	0.2308	0.2737	0.2199	0.2147	0.264	0.4592	0.3586	0.1108	0.4188	0.3689	0.3695	0.2709	0.2938	0.3475	0.3076	0.1172	0.1465	0.0066	1.000	0.000	0.000	0.000	0.000	0.000	0.000	0.000	0.000	0.000	0.000	0.000	0.000	0.000	0.000	0.000	0.000	0.000	0.000	0.000
A_16	0.2166	0.2198	0.213	0.2652	0.3894	0.3973	0.2642	0.3953	0.3184	0.1806	0.4498	0.3388	0.3284	0.2523	0.1795	0.2691	0.1972	0.271	0.138	0.2802	0.3141	1.000	0.000	0.000	0.000	0.000	0.001	0.003	0.001	0.000	0.000	0.000	0.001	0.001	0.000	0.000	0.001	0.000	0.000	0.000
A_18	0.2478	0.2525	0.2361	0.2983	0.2629	0.2639	0.1922	0.4345	0.2095	0.0379	0.4262	0.333	0.3227	0.1674	0.2841	0.3718	0.2479	0.2354	0.1839	0.2147	0.2276	0.3148	1.000	0.000	0.000	0.000	0.000	0.000	0.000	0.000	0.000	0.000	0.000	0.000	0.000	0.000	0.000	0.000	0.000	0.000
A_03	0.1965	0.1842	0.1967	0.2868	0.2586	0.2849	0.2276	0.4257	0.3333	0.1288	0.4158	0.2689	0.2553	0.2688	0.2111	0.2963	0.2049	0.2466	0.1626	0.2184	0.2918	0.307	0.2617	1.000	0.952	0.978	0.000	0.000	0.018	0.015	0.006	0.000	0.007	0.001	0.000	0.001	0.004	0.002	0.002	0.000
A_58	0.2655	0.2664	0.2507	0.3532	0.3086	0.3464	0.2865	0.4562	0.4097	0.2	0.5027	0.2728	0.2739	0.2813	0.2557	0.3447	0.2472	0.2985	0.2111	0.3173	0.3684	0.3591	0.314	0.004	1.000	0.902	0.000	0.000	0.002	0.001	0.003	0.000	0.003	0.001	0.000	0.001	0.004	0.001	0.002	0.000
A_68	0.2581	0.2448	0.2529	0.326	0.2938	0.3308	0.2508	0.4169	0.3592	0.181	0.4299	0.2968	0.3024	0.2766	0.2111	0.3176	0.2306	0.3261	0.2044	0.3026	0.3584	0.3356	0.3022	0	0.0217	1.000	0.000	0.000	0.012	0.019	0.013	0.000	0.002	0.000	0.000	0.001	0.001	0.000	0.002	0.000
A_67	0.2309	0.2381	0.2521	0.3002	0.3038	0.3102	0.1935	0.3591	0.3065	0.1126	0.4027	0.3337	0.3134	0.2162	0.1838	0.2446	0.1607	0.2047	0.1167	0.2477	0.2725	0.2152	0.2275	0.2707	0.3018	0.3166	1.000	0.952	0.001	0.000	0.000	0.000	0.019	0.000	0.000	0.000	0.001	0.000	0.000	0.000
A_04	0.2603	0.2514	0.268	0.3313	0.3616	0.3618	0.2471	0.3849	0.3509	0.156	0.4313	0.3166	0.2927	0.2716	0.1975	0.2527	0.1345	0.2401	0.138	0.2793	0.3116	0.2066	0.2842	0.2877	0.3242	0.3438	0.0066	1.000	0.000	0.000	0.000	0.000	0.005	0.000	0.000	0.000	0.001	0.000	0.000	0.000
A_59	0.252	0.2388	0.3094	0.3521	0.4025	0.4242	0.3233	0.4805	0.4735	0.2562	0.4299	0.4162	0.4525	0.3723	0.2597	0.3441	0.2772	0.3718	0.2701	0.3725	0.4239	0.4106	0.4217	0.2667	0.3465	0.3178	0.3056	0.3418	1.000	0.998	0.848	0.000	0.008	0.001	0.000	0.001	0.001	0.000	0.000	0.000
A_02	0.2404	0.2366	0.312	0.3609	0.3878	0.4123	0.3002	0.4725	0.4292	0.2361	0.4292	0.3975	0.4292	0.3419	0.2602	0.3419	0.2703	0.3563	0.2661	0.3657	0.4253	0.4065	0.381	0.2222	0.2938	0.2732	0.2854	0.3247	-0.0431	1.000	0.971	0.000	0.012	0.001	0.000	0.001	0.001	0.000	0.000	0.000
A_65	0.2778	0.2843	0.3716	0.4222	0.46	0.4921	0.3875	0.5556	0.5297	0.3092	0.4988	0.4433	0.5024	0.391	0.3421	0.4402	0.3672	0.4341	0.3437	0.43	0.4742	0.4701	0.4617	0.2952	0.37	0.3363	0.3912	0.434	0.0199	0.0044	1.000	0.000	0.001	0.000	0.000	0.000	0.001	0.000	0.000	0.000
A_56	0.324	0.2979	0.2164	0.2329	0.3314	0.291	0.2408	0.3166	0.3405	0.2112	0.3306	0.3335	0.3454	0.3588	0.2293	0.2646	0.2486	0.2845	0.1545	0.3146	0.3289	0.3648	0.3375	0.3481	0.399	0.3651	0.3392	0.3684	0.3949	0.3858	0.46	1.000	0.000	0.000	0.000	0.000	0.000	0.000	0.000	0.000
A_63	0.1619	0.1439	0.1425	0.223	0.29	0.3153	0.2219	0.3352	0.3374	0.1255	0.3775	0.2204	0.2117	0.2116	0.1209	0.2527	0.1036	0.2313	0.1152	0.284	0.3382	0.2104	0.2551	0.1817	0.1787	0.1817	0.1572	0.1762	0.229	0.2138	0.2828	0.3207	1.000	0.160	0.324	0.554	0.001	0.002	0.001	0.000
A_05	0.2856	0.2658	0.2243	0.2765	0.3014	0.3114	0.2717	0.341	0.3897	0.2039	0.3637	0.2865	0.2657	0.2788	0.1329	0.2492	0.1679	0.3393	0.128	0.3452	0.3726	0.2637	0.333	0.247	0.275	0.214	0.2506	0.2769	0.3586	0.3473	0.4119	0.3299	0.0717	1.000	0.147	0.611	0.001	0.000	0.000	0.000
A_52	0.2103	0.2082	0.1811	0.2421	0.2883	0.3146	0.2769	0.3453	0.3469	0.2009	0.3693	0.2171	0.2055	0.2447	0.1271	0.2469	0.1533	0.2787	0.1555	0.3068	0.3521	0.2226	0.2791	0.1749	0.1691	0.1448	0.2157	0.2372	0.2748	0.2618	0.3047	0.3325	0.0118	0.0244	1.000	0.954	0.000	0.000	0.000	0.000
A_06	0.2976	0.2837	0.2723	0.3333	0.3479	0.3709	0.3171	0.4268	0.4557	0.2341	0.445	0.3277	0.3309	0.2703	0.1632	0.3159	0.236	0.3573	0.1745	0.3866	0.4216	0.3125	0.3618	0.2672	0.2859	0.2303	0.2881	0.3222	0.3481	0.3373	0.4037	0.4058	0.0639	0.0222	-0.031	1.000	0.001	0.000	0.000	0.000
A_60	0.2966	0.2593	0.2263	0.2842	0.2776	0.2581	0.1974	0.3498	0.3653	0.096	0.3802	0.2274	0.2147	0.2583	0.165	0.2104	0.1244	0.2338	0.1142	0.296	0.3314	0.3249	0.3011	0.2519	0.2899	0.2768	0.2489	0.2425	0.3488	0.3288	0.4064	0.283	0.2169	0.2966	0.2478	0.3243	1.000	0.038	0.030	0.000
A_62	0.3034	0.2562	0.2299	0.2889	0.3465	0.3387	0.3597	0.4675	0.4899	0.1819	0.5128	0.2918	0.2796	0.4097	0.2648	0.3582	0.2286	0.3092	0.1465	0.3143	0.2951	0.3431	0.3805	0.2738	0.3391	0.3333	0.3441	0.3097	0.477	0.471	0.5526	0.3744	0.3005	0.3422	0.3103	0.4195	0.2575	1.000	0.000	0.000
A_08	0.3415	0.3254	0.254	0.3046	0.5	0.4895	0.3333	0.4437	0.5368	0.2924	0.5147	0.4241	0.4677	0.4207	0.3643	0.4578	0.3167	0.4719	0.3032	0.4931	0.4847	0.4667	0.4348	0.482	0.4959	0.4694	0.4547	0.4794	0.5005	0.4879	0.5618	0.3543	0.3245	0.4069	0.3466	0.4586	0.3765	0.538	1.000	0.000
A_70	0.2116	0.223	0.155	0.2166	0.2856	0.2883	0.1882	0.3318	0.3036	0.0823	0.3257	0.1843	0.1769	0.2167	0.1933	0.2774	0.1537	0.2408	0.1545	0.24	0.2486	0.2437	0.1929	0.2497	0.277	0.244	0.1856	0.2257	0.3555	0.3425	0.3797	0.2858	0.1709	0.2123	0.1806	0.246	0.1991	0.2883	0.3119	1.000
