# Supplementary material for: Divide and conquer: Multicolonial structure, nestmate recognition, and antagonistic behaviors in dense populations of the invasive ant Brachymyrmex patagonicus
Source: Ecol Evol. 2021 Mar 18;11(9):4874–86. doi: 10.1002/ece3.7396 (PMC8093738; doi:10.1002/ece3.7396)
Supplement: Supplementary file 4 — Figure S4 [file ECE3-11-4874-s006.pdf]

	C63_w01	C63_w02	C63_w03	C63_w04	C65_w01	C65_w02	C65_w03	C65_w04	C75_w01	C75_w02	C75_w03	C75_w04	A54_w01	A54_w02	A54_w03	A54_w04	A56_w01	A56_w02	A56_w03	A56_w04	A52_w01	A52_w02	A52_w03	A52_w04	A70_w01	A70_w02	A70_w03	A70_w04	A69_w01	A69_w02	A69_w03	A69_w04
C63_w01	0.00	2.57	5.06	3.64	6.82	6.36	7.29	7.11	7.24	8.00	6.39	6.59	7.56	5.55	6.47	6.44	8.13	7.18	8.21	8.15	8.07	7.90	6.91	6.98	5.83	4.84	5.31	5.82	6.23	4.45	4.25	4.98
C63_w02	2.57	0.00	6.37	4.38	7.73	7.56	8.19	7.82	8.31	8.68	7.40	7.01	8.46	6.50	7.39	6.97	8.95	8.03	9.10	8.55	9.28	9.09	8.10	8.02	6.27	5.27	5.92	6.62	7.79	5.44	3.78	6.06
C63_w03	5.06	6.37	0.00	3.83	5.81	5.18	5.73	6.18	6.35	7.21	4.89	6.24	7.77	6.23	5.46	6.58	7.41	6.19	7.41	7.67	6.03	5.61	4.50	4.67	5.59	4.37	4.13	3.42	5.73	4.98	6.24	4.46
C63_w04	3.64	4.38	3.83	0.00	6.24	5.72	6.38	6.30	6.57	6.99	5.34	5.38	7.60	6.01	5.78	5.87	7.55	6.51	7.79	7.67	7.29	7.00	5.39	5.59	5.45	3.64	4.09	4.85	6.27	3.39	4.83	4.29
C65_w01	6.82	7.73	5.81	6.24	0.00	2.19	2.68	2.93	6.93	6.43	4.87	6.34	4.73	4.63	4.50	6.29	5.53	5.00	4.93	6.46	5.38	4.89	5.82	4.21	6.84	5.81	5.23	4.81	8.08	7.39	7.85	6.06
C65_w02	6.36	7.56	5.18	5.72	2.19	0.00	3.12	3.16	6.60	6.46	4.73	6.24	4.77	4.57	4.22	6.16	5.11	4.57	4.84	6.12	5.62	5.00	5.22	4.39	6.88	5.82	5.13	4.71	7.28	6.86	7.55	5.59
C65_w03	7.29	8.19	5.73	6.38	2.68	3.12	0.00	2.20	6.82	6.74	4.68	6.11	5.79	5.54	3.92	6.26	5.65	5.60	5.97	7.24	5.05	4.51	5.51	4.16	6.05	5.64	4.86	4.39	7.91	7.29	8.22	6.07
C65_w04	7.11	7.82	6.18	6.30	2.93	3.16	2.20	0.00	7.59	6.62	5.04	6.15	5.90	5.71	3.97	5.84	5.89	5.99	6.35	7.42	6.30	5.75	6.13	5.32	6.53	5.84	5.20	5.05	8.34	7.43	7.83	6.30
C75_w01	7.24	8.31	6.35	6.57	6.93	6.60	6.82	7.59	0.00	6.57	4.92	5.26	8.28	7.30	7.12	8.61	8.05	7.64	7.93	7.17	7.73	7.43	7.54	5.66	6.65	6.68	6.16	5.93	5.22	5.64	7.41	4.81
C75_w02	8.00	8.68	7.21	6.99	6.43	6.46	6.74	6.62	6.57	0.00	3.83	5.01	7.55	7.20	6.05	6.60	6.90	7.28	7.14	6.70	7.90	7.44	7.82	5.97	7.75	7.25	6.79	6.47	8.35	6.92	8.04	5.46
C75_w03	6.39	7.40	4.89	5.34	4.87	4.73	4.68	5.04	4.92	3.83	0.00	3.13	6.28	5.79	4.02	5.63	5.32	5.37	5.94	6.19	5.69	5.29	5.78	3.45	6.16	5.25	4.78	4.28	6.60	5.32	6.47	3.29
C75_w04	6.59	7.01	6.24	5.38	6.34	6.24	6.11	6.15	5.26	5.01	3.13	0.00	7.36	6.54	5.57	6.10	6.92	7.03	7.48	7.32	7.83	7.39	7.43	5.39	6.56	5.66	5.56	5.91	6.69	4.80	5.28	3.06
A54_w01	7.56	8.46	7.77	7.60	4.73	4.77	5.79	5.90	8.28	7.55	6.28	7.36	0.00	3.12	4.40	5.86	5.34	5.26	4.27	6.68	6.71	6.28	7.52	6.39	7.99	7.19	6.52	6.54	9.31	8.71	8.60	7.26
A54_w02	5.55	6.50	6.23	6.01	4.63	4.57	5.54	5.71	7.30	7.20	5.79	6.54	3.12	0.00	4.11	4.45	6.48	5.97	5.75	6.72	6.43	5.92	6.62	6.05	6.70	5.96	5.57	5.69	7.56	6.97	7.04	5.97
A54_w03	6.47	7.39	5.46	5.78	4.50	4.22	3.92	3.97	7.12	6.05	4.02	5.57	4.40	4.11	0.00	4.12	4.98	5.20	5.55	6.82	5.22	4.52	5.41	4.83	6.15	5.50	4.75	4.38	7.87	7.05	7.43	5.59
A54_w04	6.44	6.97	6.58	5.87	6.29	6.16	6.26	5.84	8.61	6.60	5.63	6.10	5.86	4.45	4.12	0.00	7.40	7.25	7.92	7.74	6.88	6.40	6.25	6.81	7.20	6.13	6.10	6.40	8.61	6.63	7.04	5.99
A56_w01	8.13	8.95	7.41	7.55	5.53	5.11	5.65	5.89	8.05	6.90	5.32	6.92	5.34	6.48	4.98	7.40	0.00	2.42	2.88	4.73	6.61	6.27	7.12	5.58	7.45	6.45	5.86	5.64	9.69	8.61	8.85	7.12
A56_w02	7.18	8.03	6.19	6.51	5.00	4.57	5.60	5.99	7.64	7.28	5.37	7.03	5.26	5.97	5.20	7.25	2.42	0.00	2.75	4.26	5.81	5.58	6.05	4.89	6.89	5.50	5.02	4.74	9.16	7.95	8.08	6.56
A56_w03	8.21	9.10	7.41	7.79	4.93	4.84	5.97	6.35	7.93	7.14	5.94	7.48	4.27	5.75	5.55	7.92	2.88	2.75	0.00	4.69	6.91	6.48	7.66	5.75	7.60	6.68	5.93	5.64	9.71	9.07	9.01	7.38
A56_w04	8.15	8.55	7.67	7.67	6.46	6.12	7.24	7.42	7.17	6.70	6.19	7.32	6.68	6.72	6.82	7.74	4.73	4.26	4.69	0.00	7.97	7.58	7.90	6.66	7.44	6.58	6.16	5.96	9.67	8.46	8.48	7.24
A52_w01	8.07	9.28	6.03	7.29	5.38	5.62	5.05	6.30	7.73	7.90	5.69	7.83	6.71	6.43	5.22	6.88	6.61	5.81	6.91	7.97	0.00	1.30	3.52	3.92	7.98	7.20	6.75	5.82	8.46	8.12	9.36	6.78
A52_w02	7.90	9.09	5.61	7.00	4.89	5.00	4.51	5.75	7.43	7.44	5.29	7.39	6.28	5.92	4.52	6.40	6.27	5.58	6.48	7.58	1.30	0.00	3.32	3.76	7.75	6.95	6.40	5.38	8.22	7.96	9.05	6.47
A52_w03	6.91	8.10	4.50	5.39	5.82	5.22	5.51	6.13	7.54	7.82	5.78	7.43	7.52	6.62	5.41	6.25	7.12	6.05	7.66	7.90	3.52	3.32	0.00	4.40	7.68	6.39	6.11	5.53	7.24	6.47	8.07	5.93
A52_w04	6.98	8.02	4.67	5.59	4.21	4.39	4.16	5.32	5.66	5.97	3.45	5.39	6.39	6.05	4.83	6.81	5.58	4.89	5.75	6.66	3.92	3.76	4.40	0.00	6.65	5.58	5.05	4.26	6.92	6.29	7.68	4.76
A70_w01	5.83	6.27	5.59	5.45	6.84	6.88	6.05	6.53	6.65	7.75	6.16	6.56	7.99	6.70	6.15	7.20	7.45	6.89	7.60	7.44	7.98	7.75	7.68	6.65	0.00	3.01	2.60	3.61	7.60	6.16	6.89	6.09
A70_w02	4.84	5.27	4.37	3.64	5.81	5.82	5.64	5.84	6.68	7.25	5.25	5.66	7.19	5.96	5.50	6.13	6.45	5.50	6.68	6.58	7.20	6.95	6.39	5.58	3.01	0.00	1.57	3.07	7.60	5.00	5.80	5.26
A70_w03	5.31	5.92	4.13	4.09	5.23	5.13	4.86	5.20	6.16	6.79	4.78	5.56	6.52	5.57	4.75	6.10	5.86	5.02	5.93	6.16	6.75	6.40	6.11	5.05	2.60	1.57	0.00	2.00	7.26	5.43	6.17	5.10
A70_w04	5.82	6.62	3.42	4.85	4.81	4.71	4.39	5.05	5.93	6.47	4.28	5.91	6.54	5.69	4.38	6.40	5.64	4.74	5.64	5.96	5.82	5.38	5.53	4.26	3.61	3.07	2.00	0.00	7.22	6.12	6.76	5.16
A69_w01	6.23	7.79	5.73	6.27	8.08	7.28	7.91	8.34	5.22	8.35	6.60	6.69	9.31	7.56	7.87	8.61	9.69	9.16	9.71	9.67	8.46	8.22	7.24	6.92	7.60	7.60	7.26	7.22	0.00	4.80	6.81	4.71
A69_w02	4.45	5.44	4.98	3.39	7.39	6.86	7.29	7.43	5.64	6.92	5.32	4.80	8.71	6.97	7.05	6.63	8.61	7.95	9.07	8.46	8.12	7.96	6.47	6.29	6.16	5.00	5.43	6.12	4.80	0.00	4.80	3.52
A69_w03	4.25	3.78	6.24	4.83	7.85	7.55	8.22	7.83	7.41	8.04	6.47	5.28	8.60	7.04	7.43	7.04	8.85	8.08	9.01	8.48	9.36	9.05	8.07	7.68	6.89	5.80	6.17	6.76	6.81	4.80	0.00	4.24
A69_w04	4.98	6.06	4.46	4.29	6.06	5.59	6.07	6.30	4.81	5.46	3.29	3.06	7.26	5.97	5.59	5.99	7.12	6.56	7.38	7.24	6.78	6.47	5.93	4.76	6.09	5.26	5.10	5.16	4.71	3.52	4.24	0.00

Figure S3: Matrice of chemical differentiation between each pair of individuals
