# Supplementary material for: Divide and conquer: Multicolonial structure, nestmate recognition, and antagonistic behaviors in dense populations of the invasive ant Brachymyrmex patagonicus
Source: Ecol Evol. 2021 Mar 18;11(9):4874–86. doi: 10.1002/ece3.7396 (PMC8093738; doi:10.1002/ece3.7396)
Supplement: Supplementary file 5 — Figure S5 [file ECE3-11-4874-s002.pdf]

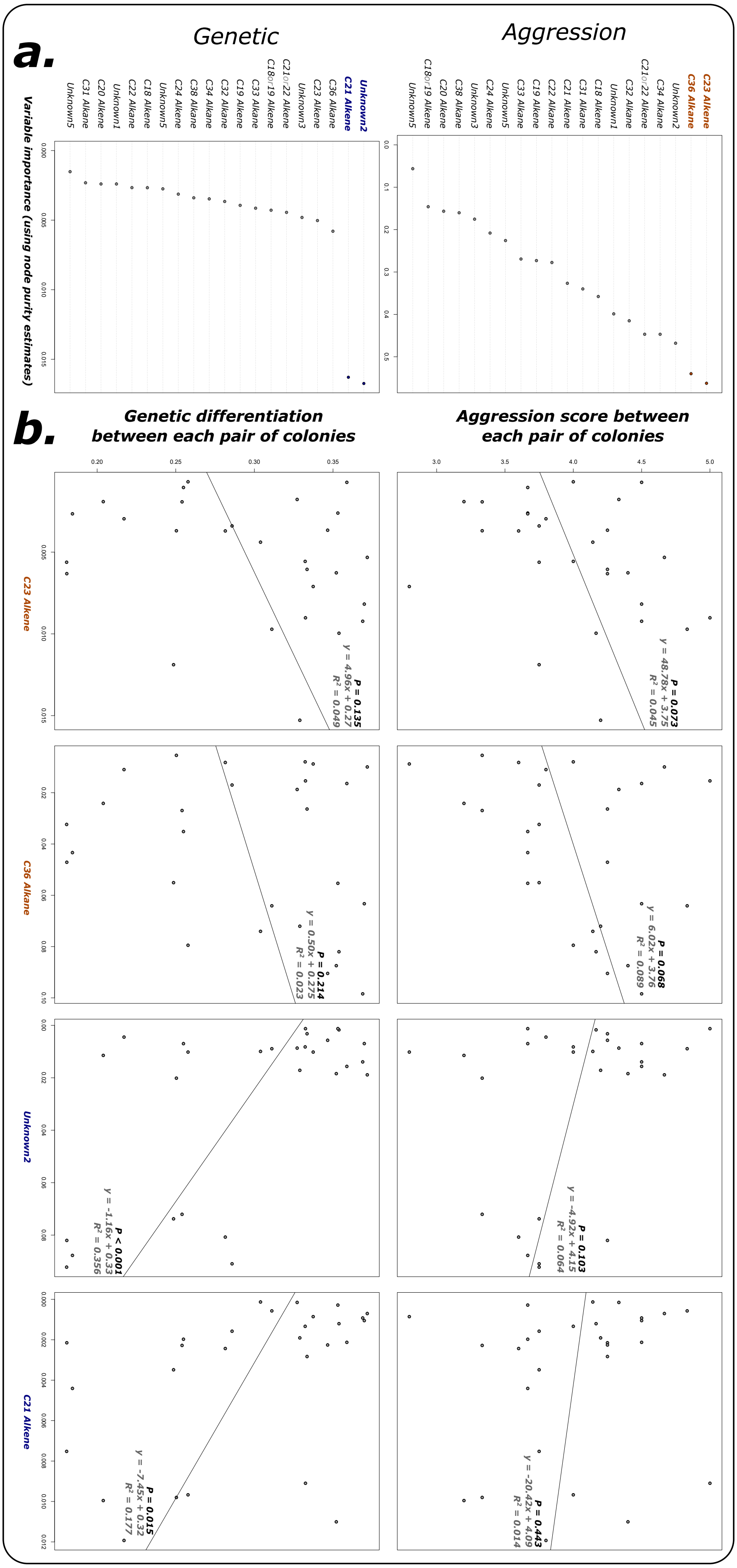
a.
Genetic
Aggression
Variable importance (using node purity estimates)
Unknown5
C31 Alkane
C20 Alkene
Unknown1
C22 Alkane
C18 Alkane
Unknown5
C24 Alkane
C38 Alkane
C34 Alkane
C32 Alkane
C19 Alkene
C33 Alkane
C18 or 19 Alkene
C21 or 22 Alkene
Unknown3
C23 Alkane
C36 Alkane
C21 Alkene
Unknown2
C23 Alkene
C36 Alkane
b.
Genetic differentiation between each pair of colonies
Aggression score between each pair of colonies
C23 Alkene
C36 Alkane
Unknown2
C21 Alkene
P = 0.135
y = 4.96x + 0.27
R² = 0.049
P = 0.073
y = 48.78x + 3.75
R² = 0.045
P = 0.214
y = 0.50x + 0.275
R² = 0.023
P = 0.068
y = 6.02x + 3.76
R² = 0.089
P < 0.001
y = -1.16x + 0.33
R² = 0.356
P = 0.103
y = -4.92x + 4.15
R² = 0.064
P = 0.015
y = -7.45x + 0.32
R² = 0.177
P = 0.443
y = -20.42x + 4.09
R² = 0.014
