# Supplementary material for: Divide and conquer: Multicolonial structure, nestmate recognition, and antagonistic behaviors in dense populations of the invasive ant Brachymyrmex patagonicus
Source: Ecol Evol. 2021 Mar 18;11(9):4874–86. doi: 10.1002/ece3.7396 (PMC8093738; doi:10.1002/ece3.7396)
Supplement: Supplementary file 6 — Table S1 [file ECE3-11-4874-s003.pdf]

Trail	Number of worker genotyped	Relatedness	Collection Period	Colony ID for trails sampled during collection period 2.
A_01	4	0.63	2	same as 53
A_02	4	0.69	2	same as 59
A_03	4	0.65	2	same as 58
A_04	4	0.71	2	same as 67
A_05	4	0.51	2	same as 52
A_06	3	0.68	2	same as 63
A_07	4	0.81	2	new
A_08	4	0.77	2	new
A_09	4	0.24	2	new
A_10	4	0.85	2	same as 64
A_11	4	0.39	2	new
A_13	4	0.18	2	new, same as 15
A_14	4	0.83	2	same as 69
A_15	4	0.95	2	new, same as 13
A_16	4	0.79	2	new
A_17	4	0.66	2	same as 57
A_18	5	0.76	2	new
A_19	4	0.75	2	same as 54
A_20	4	0.36	2	same but distant from 51
A_51	4	0.67	1	
A_52	8	0.41	1	
A_53	4	0.50	1	
A_54	8	0.78	1	
A_55	4	0.86	1	
A_56	8	0.73	1	
A_57	4	0.63	1	
A_58	4	0.78	1	
A_59	4	0.71	1	
A_60	4	0.52	1	
A_61	4	0.84	1	
A_62	4	0.71	1	
A_63	4	0.30	1	
A_64	4	0.32	1	
A_65	4	0.73	1	
A_66	4	0.06	1	
A_67	4	0.67	1	
A_68	4	0.68	1	
A_69	8	0.65	1	
A_70	8	0.52	1	
A_71	4	-0.03	1	
C_01	4	0.68	2	same as 66
C_02	4	0.74	2	same as 61
C_03	6	0.48	2	new

C_04	6	0.37	2	same as 51
C_05	4	0.79	2	same as 67
C_06	4	0.75	2	same as 57
C_07	6	0.47	2	same as 63
C_08	4	0.71	2	same as 62
C_09	4	0.70	2	same as 75
C_10	4	0.07	2	same as 81
C_11	4	0.71	2	same as 73
C_12	4	0.79	2	same as 65
C_13	6	0.72	2	same but distant from 85
C_14	6	0.49	2	same as 71
C_15	4	0.90	2	new
C_16	4	0.70	2	new
C_17	4	0.84	2	same as 85
C_18	5	0.20	2	new, same as 20
C_19	4	0.54	2	new
C_20	4	0.80	2	new, same as 18
C_51	4	0.66	1	
C_52	4	0.83	1	
C_53	6	0.11	1	
C_54	4	0.34	1	
C_55	4	0.72	1	
C_56	4	0.69	1	
C_57	4	0.74	1	
C_58	4	0.85	1	
C_59	4	0.70	1	
C_60	4	0.74	1	
C_61	4	0.73	1	
C_62	4	0.67	1	
C_63	8	0.73	1	
C_64	4	0.78	1	
C_65	8	0.82	1	
C_66	4	0.75	1	
C_67	4	0.72	1	
C_68	4	0.22	1	
C_69	4	0.70	1	
C_70	4	0.00	1	
C_71	4	0.71	1	
C_72	4	0.70	1	
C_73	4	0.69	1	
C_74	4	0.85	1	
C_75	8	0.68	1	
C_76	4	0.81	1	
C_77	4	0.68	1	
C_78	6	-0.01	1	
C_79	4	0.85	1	
C_80	4	0.45	1	

C_81	4	0.63	1
C_82	4	0.80	1
C_83	4	0.69	1
C_84	4	0.51	1
C_85	4	0.79	1
SUM	427		
Average	4.49	0.62	
SD	1.20	0.23	
